# Continuous Exercise but Not High Intensity Interval Training Improves Fat Distribution in Overweight Adults

**DOI:** 10.1155/2014/834865

**Published:** 2014-01-19

**Authors:** Shelley E. Keating, Elizabeth A. Machan, Helen T. O'Connor, James A. Gerofi, Amanda Sainsbury, Ian D. Caterson, Nathan A. Johnson

**Affiliations:** ^1^Discipline of Exercise and Sports Science, The University of Sydney, Lidcombe, NSW 2141, Australia; ^2^Boden Institute of Obesity, Nutrition, Exercise and Eating Disorders, The University of Sydney, Sydney, NSW 2006, Australia

## Abstract

*Objective*. The purpose of this study was to assess the effect of high intensity interval training (HIIT) versus continuous aerobic exercise training (CONT) or placebo (PLA) on body composition by randomized controlled design. *Methods*. Work capacity and body composition (dual-energy X-ray absorptiometry) were measured before and after 12 weeks of intervention in 38 previously inactive overweight adults. *Results*. There was a significant group × time interaction for change in work capacity (*P* < 0.001), which increased significantly in CONT (23.8 ± 3.0%) and HIIT (22.3 ± 3.5%) but not PLA (3.1 ± 5.0%). There was a near-significant main effect for percentage trunk fat, with trunk fat reducing in CONT by 3.1 ± 1.6% and in PLA by 1.1 ± 0.4%, but not in HIIT (increase of 0.7 ± 1.0%) (*P* = 0.07). There was a significant reduction in android fat percentage in CONT (2.7 ± 1.3%) and PLA (1.4 ± 0.8%) but not HIIT (increase of 0.8 ± 0.7%) (*P* = 0.04). *Conclusion*. These data suggest that HIIT may be advocated as a time-efficient strategy for eliciting comparable fitness benefits to traditional continuous exercise in inactive, overweight adults. However, in this population HIIT does not confer the same benefit to body fat levels as continuous exercise training.

## 1. Introduction

Epidemiological data show that the majority of the adult population fails to meet recommended physical activity levels [1]. This contributes to the global epidemic of overweight/obesity and associated cardiovascular disease. A reason often cited for failure to participate in regular exercise is a perceived lack of time [2].

High intensity interval training (HIIT) could potentially provide health benefits in a time-efficient manner. This involves repeated bursts of vigorous exercise interspersed with low intensity recovery. There is growing evidence from healthy populations that HIIT leads to a range of cardiovascular and metabolic benefits that are similar to or greater in magnitude than those achieved with regular continuous aerobic exercise. These benefits include increased cardiorespiratory fitness [3–9] and work capacity [10], increased muscle mitochondrial biogenesis and GLUT-4 levels [11], and improved insulin sensitivity [4, 6, 7]. Relative to the effect of continuous aerobic exercise, HIIT has also been shown to induce comparable improvements in fitness and insulin sensitivity in clinical populations, including those with overweight/obesity [12–17], cardiovascular disease [18–21], metabolic syndrome [17], and type 2 diabetes [22–24]. In some of these studies [7, 21, 23] the benefits of HIIT were achieved with programs requiring similar training time, but most found a benefit with 50–60% of the training time used in the traditional continuous aerobic exercise comparison group [3, 5, 8, 16, 18–20].

On the basis of these metabolic and fitness benefits, it has been argued that HIIT could be used as a time-effective therapy for the management of body fat levels in overweight and obese individuals [4, 8, 13, 14, 16, 17, 21, 23, 25–27]. However, there is currently a dearth of knowledge about the independent effects of HIIT on body composition, relative to the effects of continuous aerobic exercise training. There is some evidence that HIIT programs may reduce body mass, trunk fat, and waist circumference in adolescents and adults [4, 8, 9, 14, 17, 23–27]. However, some of these studies only compared HIIT with a nonexercising control group [25, 27] or failed to delineate the independent benefit of HIIT from the confounding influence of concurrent continuous training [23, 24] or dietary intervention [14] or employed young, healthy cohorts [4, 8, 9, 26]. In fact, much of the previous research showing positive cardiometabolic effects of HIIT was performed in apparently healthy cohorts. These used HIIT interventions that required repeated 8–30 sec bouts of “all-out” sprint exercise [4, 8, 13, 15, 26, 27]. Such explosive exercise is associated with large spikes in plasma adrenalin [28] and heart rate [27, 28] which may be maintained between 80 and 90% maximum for the duration of the exercise session [27]. Although perhaps suitable for healthy younger populations, sprint training is incongruent with the rationale for using HIIT in a clinical setting for overweight or obese individuals: not only to gain time-efficient benefits from exercise but also to avoid risks associated with high workloads [29].

Whilst HIIT programs that are of low risk have been shown to produce cardiometabolic benefits (improvements in glycaemic control, insulin sensitivity, and skeletal muscle oxidative capacity) in people with coronary artery disease and chronic obstructive pulmonary disease [18–21, 30] or in overweight/obese [12, 14] or type 2 diabetes cohorts [22], there has been no controlled comparison of the independent effect of HIIT versus that of continuous exercise training on body composition in these populations for whom an improved body fat distribution is sought.

We therefore conducted a randomized placebo-controlled trial to examine the effect of 12 weeks of HIIT versus continuous aerobic exercise versus a sham-exercise placebo control on body composition and cardiovascular risk factors in overweight, previously inactive adults. We hypothesized that HIIT would improve fitness and reduce body fat, trunk fat, and android fat and that these benefits would be comparable to those achieved with traditional continuous exercise training and could be achieved in 50–60% of the total time.

## 2. Subjects and Methods

### 2.1. Participants (Eligibility, Settings, and Locations)

38 inactive (exercising < 3 days/week) and overweight (BMI 25 to 29.9) adult (18- to 55-year-old) men (*n* = 7) and women (*n* = 31) were randomized to receive three sessions/week for 12 weeks of regular HIIT, continuous moderate intensity exercise (CONT), or placebo exercise (PLA) intervention. Participants were recruited between June 2010 and October 2012 via university noticeboards, electronic bulletins, and clinical trial databases. The study was approved by the Human Research Ethics Committee of The University of Sydney and subjects provided written informed consent after obtaining clearance from a medical practitioner to undertake the study. Volunteers were excluded if taking lipid-lowering medication or if they had evidence of any medical disorder not fully controlled or with varying medications, diabetes, or hypertension. Two participants were taking antihypertensive medication with the dose unchanged for >12 months. Medication dose was not altered throughout the study and compliance was checked verbally each week.

**Figure 1:**
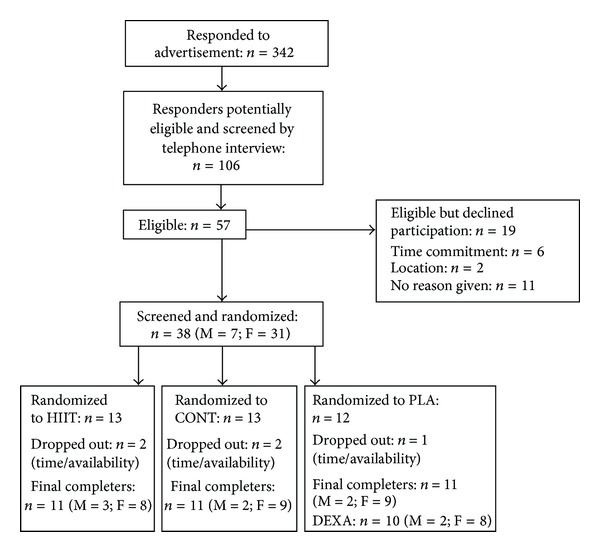
Flowchart showing the study process. M: male, F: female, HIIT: high intensity interval training, CONT: continuous aerobic exercise, PLA: placebo control group.

106 individuals were screened by telephone interview with 38 eligible volunteers undergoing initial assessment and randomization. Five participants did not complete the program (Figure 1). There was no significant difference between completers and noncompleters and reasons for dropout were unrelated to the study interventions and classified as “missing completely at random” [31]. The majority of participants were Caucasian (*n* = 25), with the remainder Asian (*n* = 3), Indian (*n* = 2), Arabic (*n* = 2), European (*n* = 2), West Indian (*n* = 1), South American (*n* = 1), and Hispanic (*n* = 1).

Volunteers were required to abstain from alcohol, over-the-counter medication, and strenuous exercise for 24 hours prior to baseline and postintervention testing. Randomization was undertaken after baseline assessments by equally distributed pregenerated list (http://www.randomization.com/) of permuted blocks. Primary outcomes were change in cardiorespiratory fitness/work capacity and body fat distribution. Secondary outcomes were anthropometric measures (body weight, body mass index (BMI), and waist and hip circumference), blood lipids, serum biochemistry, and resting blood pressure.

### 2.2. Cardiorespiratory Fitness/Work Capacity

Cardiorespiratory fitness/work capacity was assessed by graded maximal exercise test on an electronically braked cycle ergometer (Lode Corival, The Netherlands) under the supervision of the study physician. After a 3-minute warm-up at 35 W and 65 W for women and men, respectively, intensity was increased by 25 W every 150 seconds until volitional fatigue. Heart rate, blood pressure, and 12-lead ECG were recorded at each stage of exercise and participants were verbally encouraged to perform to volitional fatigue. Rating of perceived exertion (RPE) was measured using the Borg scale [32]. The test was terminated when the pedalling rate fell below 50 revolutions per minute or the participant ceased exercise. Peak work capacity (*W*_peak_) was measured [33] and VO_2peak_ estimated [34].

### 2.3. Body Composition, Anthropometrics, and Blood Pressure

Total body and regional fat distributions were measured by dual-energy X-ray absorptiometry (DEXA) (Lunar Prodigy, GE Medical Systems, Madison, WI, USA, software enCORE 2011 Version 13.60.033). The trunk region included the bottom of the neck line to the top of the pelvis, excluding the upper limbs. The android region (which incorporates abdominal subcutaneous and visceral adipose tissue) included the cut of the pelvic region to 20% of the distance between the pelvic cut and the bottom of the neck line, excluding the arms. The gynoid measure included the height equal to 2 times the height of the android region (Lunar enCORE-based X-ray Bone Densitometry User Manual Revision 6, September 2010). DEXA scanning and analyses were performed by an individual who was blinded to group allocation. Stature was measured to the nearest 0.1 cm by stadiometer (Tanita Best Weight, Seca Model 220, Germany). Waist circumference was measured in the horizontal plane, midway between the inferior margin of the ribs and the superior border of the iliac crest in deep expiration [33]. Blood pressure was measured manually from each arm after 10–15 minutes of quiet sitting with a second reading taken when there was a difference >10 mmHg.

### 2.4. Blood Sampling and Analysis

Venous blood (8 mL) was collected from the antecubital vein after an overnight fast (>10 hrs) into 2 serum separation tubes. The whole blood sample was stored at 4°C for 2-3 h prior to analysis by an accredited commercial laboratory (Douglass Hanly Moir Pty Ltd., Sydney, Australia). Analysis was performed on the same day as that of collection of serum glucose, insulin and lipids (including triglycerides (TAG), total cholesterol (TC), high density lipoprotein cholesterol (HDL-C), and low density lipoprotein cholesterol (LDL-C)), alanine aminotransferase (ALT), aspartate aminotransferase (AST), and high sensitivity C-reactive protein (hs-CRP). The other tube of blood was used for determination of serum free fatty acids (FFAs). These tubes were centrifuged at room temperature at 4000 g and serum was stored at −20°C before analysis.

Results from the screening visit were used to determine the presence of metabolic syndrome using the International Diabetes Federation definition [35].

### 2.5. Habitual Physical Activity and Dietary Control

Participants were asked to maintain their habitual activity and eating behaviours for the duration of the intervention. Mean time spent in sedentary time, physical activity, steps per day, and daily energy expenditure were analysed by a triaxial accelerometer worn on the upper arm, which also estimated energy expenditure through galvanic skin response and heat flux (SenseWear, BodyMedia Inc., PA, USA) for three nonexercising days (two weekdays and one weekend day) during weeks 1 and 12. Accelerometers were worn for 24 h/day except during water-based activities such as showering. Participants also completed a diet diary and subjective physical activity questionnaire [36] during this period. Diet diaries were analysed by a dietitian who was blinded to group allocation, and average daily intake of energy and macronutrients was quantified by Foodworks (Foodworks 2009, Xyris Software, v6.0.6502). Accelerometer data were analysed by an assessor blinded to group allocation and values were averaged over 24 h. Data were omitted from analysis if the monitor was worn for <90% of a 24-hour period.

### 2.6. Exercise Intervention

All exercise training in the intervention groups was supervised by an accredited exercise physiologist. Heart rate, RPE, and blood pressure were continuously monitored throughout training. Although it was not possible to be blinded to exercise group allocation, participants were blinded to the primary purpose of the study and the placebo group were instructed that the stretching/massage/fitball intervention was intended to reduce inflammation and body fat.

*High Intensity Interval Training (HIIT).* The progressive HIIT program consisted of repeated bursts of exercise on the cycle ergometer at a power output designed to elicit 120% of VO_2peak_. Efforts were interspersed with cycling at a low intensity (30 W). This program was based on interventions by Little et al. [22] and others [7, 12, 14] which were undertaken in clinical populations, were well tolerated, and induced significant improvements in fitness [12, 14, 22] and insulin sensitivity [12, 22]. For all participants intervals were progressed over the first four weeks from four intervals on a work : recovery schedule of 30–45 seconds at 120% of VO_2peak_ : 120–180 seconds at low intensity. For weeks 5–12 (i.e., the majority of the intervention period) all participants performed six intervals on a work : recovery schedule of 60 : 120 seconds (Table 1). When combined with a six-minute warm-up/cooldown, total time commitment per session ranged from 20 to 24 minutes. Participants undertook exercise on three days each week (60 to 72 min/week total training time). 

*Continuous Endurance Training (CONT).* The CONT program involved continuous cycling on the ergometer. In accordance with current recommendations [37], training was progressed from 30 minutes at an intensity of 50% of VO_2peak_ in week one to 45 minutes at an intensity of 65% of VO_2peak_ by the fifth week of the study (Table 1). Including warm-up/cooldown, total time commitment per session ranged from 36 to 48 minutes. Participants undertook exercise on three days each week (108 to 144 min/week total training time). 

*Placebo Group (PLA). *Participants in the PLA group were prescribed a stretching, self-massage, and fitball program. Participants received one fortnightly supervised session which involved instruction of new exercises to be performed for two weeks and a 5-minute cycle at very low intensity (30 W) to maintain familiarity with the cycle ergometer. When combined with home-based sessions, participants in PLA undertook the sham exercise on three days per week. During the home-based sessions, participants were instructed to warm up and cool down by walking slowly for five minutes. Sessions were recorded in a log-book to ensure compliance. The PLA intervention was designed not to elicit cardiometabolic improvements but to control for factors such as attention and participation in a lifestyle intervention.

### 2.7. Statistics

Compliance was calculated as total number of sessions attended/total number of sessions available ×100. An intention-to-treat analysis was employed with group mean change scores imputed for dropouts. This single imputation method is valid when data is assumed to be missing completely at random independent of study intervention and group allocation [31]. The mean group scores were imputed for fitness, anthropometry, and blood parameters for the two dropouts in HIIT and CONT and the one dropout in PLA. For DEXA, one individual in PLA, who completed all other measures, did not undergo the final scan and therefore mean group scores were imputed for two participants in all groups. The group × time interaction for the change score of all outcome measures was assessed by analysis of covariance (ANCOVA) using the baseline value as the covariate (SPSS version 17.0), with the Bonferroni post hoc comparison used to locate significant treatment differences. Effect size was calculated using Hedges' g corrected for bias with 95% confidence intervals. Relationships between change in android fat with intervention and change in potential confounders (diet or nonexercise physical activity time) during treatment were determined by the Pearson correlation coefficients. Statistical significance was accepted at *P* < 0.05. Values are reported as means ± SE.

## 3. Results

The study population had an average BMI of 28.3 ± 0.3 kg/m^2^, total body fat of 42.5 ± 1.1%, and mean age of 42.8 ± 1.4 years. Other participant characteristics at baseline are described in Table 2. Thirty-three of the 38 enrolled participants completed the training and placebo interventions (86%) representing 11 of 13 individuals (85%) for HIIT, 11 of 13 individuals (85%) for CONT, and 11 of 12 individuals (92%) for PLA. Postintervention dietary and habitual physical activity data were unavailable for some participants (detailed in Table 4). For individuals who completed the study, compliance with the exercise intervention was 96%, 92%, and 76% in HIIT, CONT, and PLA, respectively. There were no adverse events during testing or training in HIIT or PLA, and there was one syncopal (fainting) episode during testing in CONT. In the HIIT sessions systolic blood pressure typically increased by ~70 mmHg from rest during the high intensity intervals and subsequently reduced by ~20–30 mmHg during the recovery intervals. Heart rate typically increased by ~95 beats above resting during the high intensity intervals and subsequently reduced by ~30–40 beats during the recovery intervals. Peak systolic blood pressure (≤250 mmHg) and heart rate (≤90% HR_max_) were maintained at submaximal levels which are considered suitable for exercise training [38].

### 3.1. Cardiorespiratory Fitness and Work Capacity

There was a significant group × time interaction for change in *W*_peak_ (*P* < 0.001), which increased significantly in HIIT (22.3 ± 3.5%) and CONT (23.8 ± 3.0%) but not PLA (3.1 ± 5.0%) (Table 3). There was no significant difference in the magnitude of improvement in *W*_peak_ between HIIT and CONT (*P* = 0.86).

### 3.2. Body Mass and Body Composition

**Figure 2:**
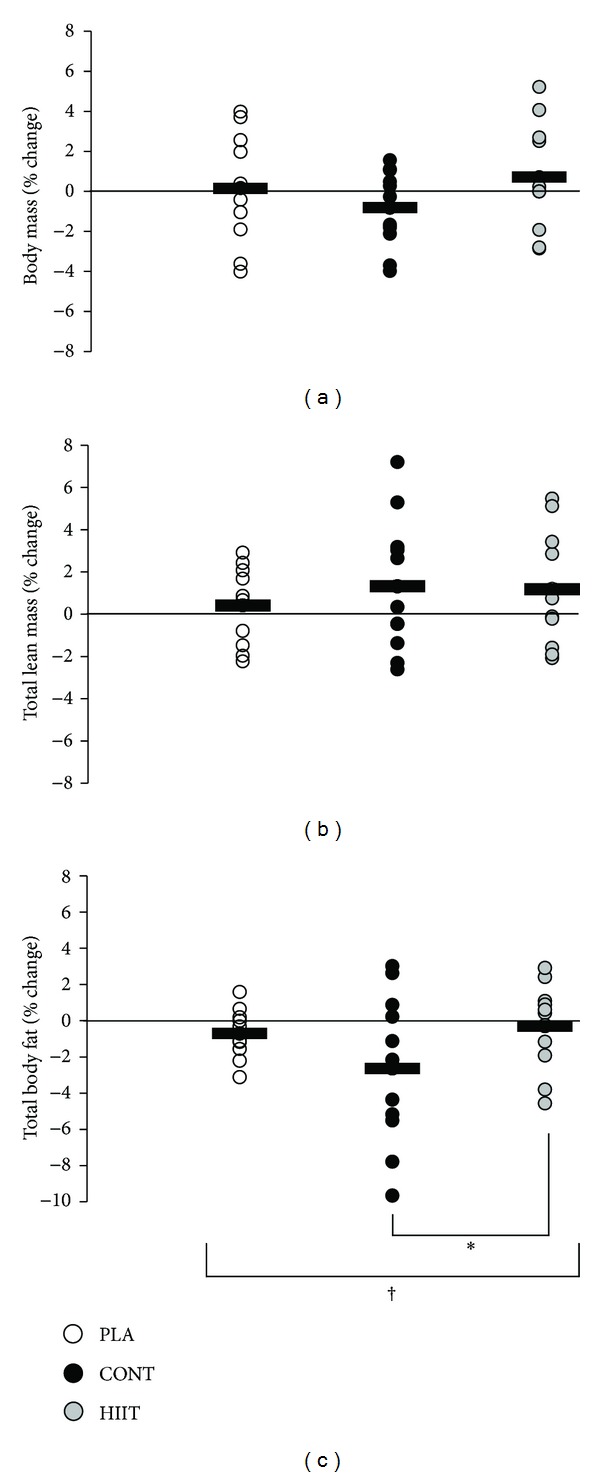
Effect of 12 weeks of high intensity interval training (HIIT) or continuous aerobic exercise (CONT) or control (PLA) on relative percent change in (a) body mass, (b) total lean mass, and (c) total body fat. Circles show individual percentage change from baseline and horizontal bars show mean group percentage change from baseline. Values are means ± SE; *n* = 13 for HIIT, *n* = 13 for CONT, and *n* = 12 for PLA. ^†^Significant treatment × time interaction (*P* < 0.05). *Significant difference between CONT and HIIT (*P* < 0.05).

**Figure 3:**
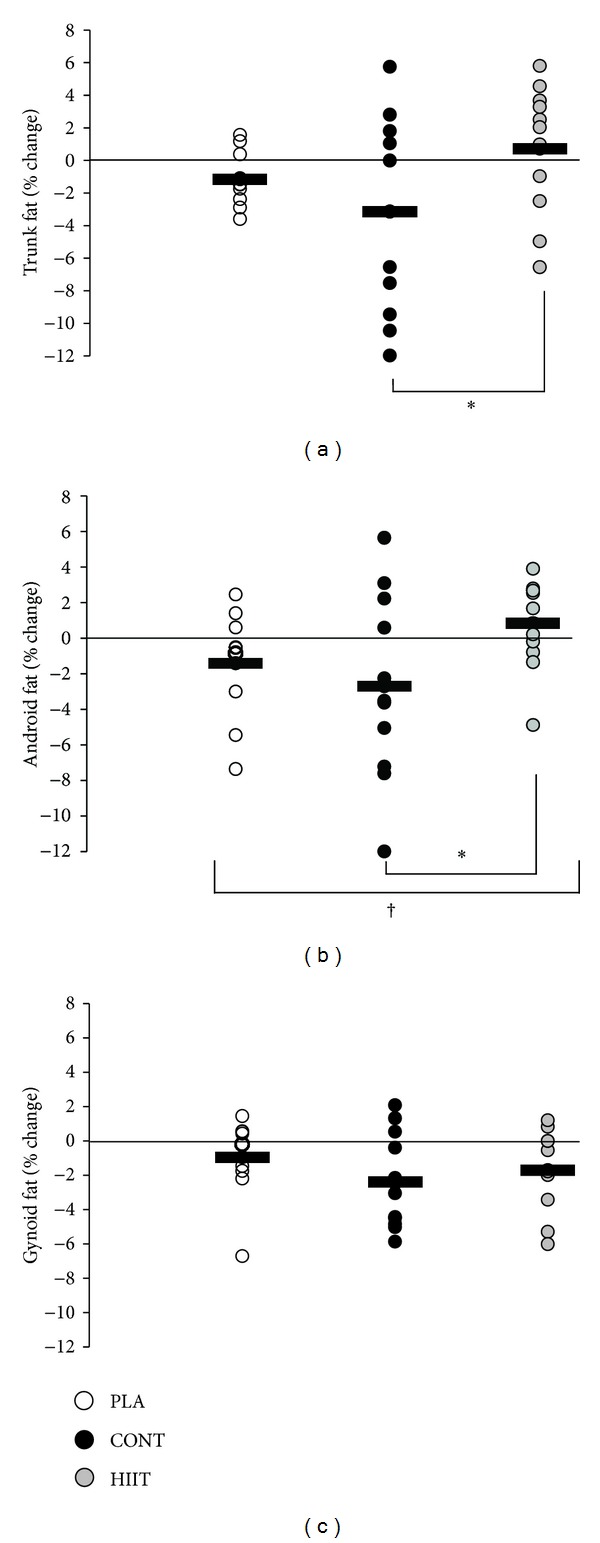
Effect of 12 weeks of high intensity interval training (HIIT) or continuous aerobic exercise (CONT) or control (PLA) on relative percent change in (a) trunk fat, (b) android fat, and (c) gynoid fat. Circles show individual percentage change from baseline and horizontal bars show mean group percentage change from baseline. Values are means ± SE; *n* = 13 for HIIT, *n* = 13 for CONT, and *n* = 12 for PLA. ^†^Significant treatment × time interaction (*P* < 0.05). *Significant difference between CONT and HIIT (*P* < 0.05).

Body mass did not change significantly in any group (*P* = 0.30) (Figure 2(a)) and there were no significant changes in lean body mass (Figure 2(b)). There was a significant main effect for total percentage body fat (*P* = 0.049) (Figure 2(c)), with body fat reducing in CONT (2.6 ± 1.1%) but not HIIT (0.3 ± 0.6%, *P* = 0.02) or PLA (0.7 ± 0.4%, *P* = 0.07). There was a near-significant main effect for percentage trunk fat (*P* = 0.07), which reduced in CONT by 3.1 ± 1.6% and PLA by 1.1 ± 0.4% but not HIIT (increase of 0.7 ± 1.0%) (Figure 3(a)). The effect of exercise on trunk fat was significantly different between HIIT and CONT (*P* = 0.02). There was a significant reduction in android fat in CONT (2.7 ± 1.3%) and PLA (1.4 ± 0.8%) but not HIIT (increase of 0.8 ± 0.6%, *P* = 0.04) (Figure 3(b)). The effect of exercise on android fat was significantly different between HIIT and CONT (*P* = 0.01). Both HIIT and CONT but not PLA tended to reduce gynoid fat relative to baseline values, but there was neither any group × time effect (*P* = 0.28) nor any significant difference between HIIT and CONT (*P* = 0.35) (Figure 3(c)).

### 3.3. Anthropometrics and Blood Pressure

There was no significant difference between groups for change in waist or hip circumference. Neither systolic nor diastolic blood pressure changed in any group (Table 3).

### 3.4. Blood Lipids and Biochemistry

There was no significant group × time interaction for changes in fasting serum AST, ALT, hs-CRP, triglycerides, HDL-C, insulin, or glucose (*P* > 0.05). There was a significant group × time interaction for total cholesterol and LDL-C (Table 3).

### 3.5. Habitual Physical Activity and Dietary Control

Accelerometry data for one participant were omitted from analysis due to not wearing the accelerometer for the required period. Another participant refused to wear the accelerometer and therefore postintervention data for habitual physical activity and energy expenditure were available for *n* = 11 in HIIT, *n* = 10 in CONT, and *n* = 10 in PLA (Table 4). Total energy expenditure, steps taken per day, time spent in sedentary behaviour, time spent in moderate physical activity, and self-reported physical activity levels were not different between groups over time (*P* > 0.05) (Table 4). Diet and physical activity diary data were unavailable for two participants in HIIT and one participant in both CONT and PLA. There was no significant group × time interaction for any measure of energy or macronutrient intake as determined by diet diaries or self-reported physical activity (*P* > 0.05) (Table 4).

### 3.6. Relationship between Change in Body Fat and Other Variables

Correlations were performed using combined data from all study participants. Change in android fat was not significantly correlated with change in daily dietary energy intake (*r* = −0.041, *P* > 0.05) or macronutrient composition (*r* = 0.095, *r* = −0.045, and *r* = 0.177 for percentage carbohydrate, fat, and protein, respectively, *P* > 0.05 for all). There was no significant correlation between change in android fat and change in daily energy expenditure (*r* = −0.025, *P* > 0.05), sedentary time (*r* = 0.002, *P* > 0.05), steps taken (*r* = −0.067), or time spent in incidental moderate activity (*r* = 0.083, *P* > 0.05).

## 4. Discussion

This is the first study to examine the efficacy of HIIT versus that of continuous aerobic exercise training on body fat levels in previously inactive, overweight adults for whom an improved fat distribution is sought. Using a 12-week randomized placebo-controlled design, we showed that HIIT resulted in a similar improvement in work capacity to that induced by continuous aerobic exercise in this cohort. Moreover, the HIIT-induced improvement in fitness was achieved in only ~50–60% of the time taken for the same gain in fitness via continuous aerobic exercise (60–72 versus 108–144 minutes per week). However, although HIIT has been suggested to be effective for the management of body fat levels [4, 8, 13, 14, 16, 17, 21, 23, 25–27], we have provided the first direct evidence that continuous aerobic exercise, but not HIIT, reduces total body fat and android fat in previously inactive, overweight adults. We thus conclude that while HIIT is a time-effective means of achieving improved fitness, it should not be promoted as a time-effective means of increasing fat loss and improving fat distribution for this population.

Our demonstration that HIIT induced a similar improvement in fitness to that of continuous aerobic exercise confirms recent reports which have examined the effect of HIIT on cardiorespiratory fitness in a range of populations [3, 4, 13, 22]. It also demonstrates that a regime involving 1 min bursts at ~maximal capacity and taking approximately 50–60% of the training time of the continuous exercise program is safe and effective. Given that aerobic fitness is a potent independent predictor of health and mortality [39], this finding provides evidence for utilising HIIT as part of the management of cardiometabolic risk in previously inactive and overweight populations. However, excess deposition of body fat, particularly abdominal fat (which includes visceral adipose tissue), is also recognized as being an independent risk factor for insulin resistance [40], cardiovascular disease [41], and death [42], and small differences in visceral adipose tissue area/volume can significantly alter risk profile [43]. Whilst the recent interest in HIIT has led to the suggestion that it may be useful for beneficial body composition change in overweight/obesity, there is a paucity of quality evidence to support this, and no randomized placebo-controlled trials in overweight cohorts have been undertaken. The clear lack of benefit of HIIT to body fat levels observed in the current study suggests that emphasizing HIIT over continuous exercise for the specific management of body fat levels in previously inactive, overweight individuals is unfounded. Until contrary evidence from randomized controlled studies is available, the current findings imply a need to reemphasise the importance of traditional continuous aerobic exercise in higher volumes which, as corroborated in the present study, has a proven ability to improve fat partitioning even in the absence of weight loss [44].

To avoid risk of cardiovascular and musculoskeletal events during training we chose a HIIT protocol that is likely to involve less risk for overweight previously sedentary people than that of an “all-out” sprinting protocol. HIIT protocols can vary in exercise intensity, timing of the work : recovery cycles, type and intensity of recovery, and the number of intervals, making comparison between studies problematic. However, generally in clinical populations (e.g., coronary artery disease, chronic obstructive pulmonary disease, type 2 diabetes, and chronic heart disease), HIIT programs use 60–240-second efforts of near-maximal or maximal aerobic exercise, with interspersed recovery periods, and have been shown to elicit a range of cardiovascular and metabolic benefits but with cardiovascular strain minimised [19–21]. For instance, in overweight [12], obese [14], and type 2 diabetes cohorts [22], 60-second bouts of exercise at near-maximal capacity, with 60–120-second recovery periods, led to significant improvements in glucose control [22], insulin sensitivity [12, 22], and skeletal muscle oxidative capacity [22]. However in all but one of these studies [14], no continuous aerobic exercise control or placebo control was included. Given our previously inactive overweight adult cohort, we employed a similar work : recovery cycle of 60 : 120 seconds. Although all participants reported maximal to near-maximal perceived exertion (Borg RPE 18–20) during the work phase, peak blood pressure (systolic blood pressure ≤ 250 mmHg) and heart rate (≤90% maximal heart rate) were within normal limits for exercise [38], and the protocol was well tolerated by participants with no adverse events. We acknowledge the need to further investigate the efficacy of HIIT interventions on fat distribution in overweight cohorts using a range of HIIT protocols.

It is now well established that regular exercise can lead to preferential fat, including visceral fat reduction (even in the absence of weight loss). Aerobic exercise significantly increases hormone-stimulated adipose lipolysis and subsequent circulating fatty acid availability, which when combined with the sustained increase in metabolic rate (VO_2_) results in increased uptake and oxidation of fatty acids in working muscle [45]. Although the precise mechanism of preferential fat reduction remains unclear, visceral adipose tissue lipolysis is believed to be particularly sensitive to these hormonal changes [46]. As acute bouts of sprint-like HIIT significantly increase the catecholamines (epinephrine and norepinephrine) and growth hormone, which stimulate lipolysis [28, 47, 48], it has been suggested that HIIT may be effective for visceral fat reduction [49]. In the absence of supporting physiological data it is difficult to speculate on the mechanism explaining the effectiveness and lack thereof of the continuous and HIIT interventions, respectively, in the current study. However, taken together, the aforementioned effects may imply that mere elevation of lipolysis and fatty acid availability via HIIT does not necessarily translate into a significant increase in fatty acid oxidation and ultimately fat loss. This may be because the total amount of fat oxidation (during and after exercise), which is a function of fatty acid availability, metabolic rate, and duration of exercise, may still be lower in HIIT than in traditional prolonged aerobic exercise.

We acknowledge that the small sample size in this study (38 enrolled, 33 completed) limits the ability to draw some conclusions about the relative potency of CONT versus that of HIIT from our trial. This affects the majority of studies in this area, as compliance with specific exercise interventions requires intensive supervision. Similarly it is difficult to definitively exclude the impact that changes in lifestyle, outside of the exercise intervention, can have on body fat outcomes in this type of research. For example there was a small reduction of android fat in PLA, and such changes are common in clinical trials under free living conditions where participants may alter their behaviour [50]. However, in our study the changes observed in the exercise interventions arguably reflect the effect of the interventions per se because there were no significant differences in dietary intake and nonexercise physical activity detected, nor were any correlations observed between these and loss of android fat. Lastly, the majority of our cohort were Caucasian females, and there were only a small number of males (*n* = 5) and a small number of people from other ethnic backgrounds. This increases the variance in our cohort and also may limit generalization of the results. Therefore, whilst our study has provided important first data comparing HIIT versus continuous aerobic exercise versus placebo in fitness and body composition in previously inactive and overweight adults, more studies are warranted in similar cohorts.

Overall, the results from this study show that continuous aerobic exercise training is effective for improving fat distribution independently of weight loss, but the HIIT intervention employed did not improve fat distribution. However, despite using 50–60% of the total training time employed in the CONT intervention, HIIT significantly improved work capacity in previously inactive and overweight adults.

## Figures and Tables

**Table 1:** Description of exercise interventions.

Week	Frequency	Intensity	Session duration		Total weekly training time (including warm-up and cooldown, min)
			Work : recovery	Intervals (number)	
HIIT					
1	3	120% VO_2peak_: 30 W	30 : 180 s	4	60
2	3	120% VO_2peak_: 30 W	30 : 120 s	5	55.5
3	3	120% VO_2peak_: 30 W	45 : 120 s	5	59.25
4	3	120% VO_2peak_: 30 W	45 : 120 s	6	67.5
5–12	3	120% VO_2peak_: 30 W	60 : 120 s	6	72
CONT					
1	3	50% VO_2peak_	30 min		108
2	3	60% VO_2peak_	40 min		138
3	3	65% VO_2peak_	45 min		144
4	3	65% VO_2peak_	45 min		144
5–12	3	65% VO_2peak_	45 min		144

HIIT: high intensity interval training; CONT: continuous aerobic exercise; W: watts; VO_2peak_: peak aerobic capacity.

**Table 2:** Baseline participant characteristics.

Characteristics	PLA (*n* = 12)	CONT (*n* = 13)	HIIT (*n* = 13)
Demographics			
Age (years)	42.9 (2.8)	44.1 (1.9)	41.8 (2.7)
Sex (*n* = M/F)	2/10	2/11	3/10
BMI (kg/m^2^)	28.2 (0.6)	28.5 (0.6)	28.2 (0.5)
Waist circumference (cm)	90.9 (3.1)	90.8 (2.1)	92.4 (2.5)
Metabolic syndrome (Y/N)	2/12	3/13	2/13
Baseline habitual physical activity (Bouchard (kJ/Kg/day))	13.8 (0.6)	13.7 (0.4)	13.4 (0.3)

Presented as mean (SE). PLA: placebo; CONT: continuous aerobic exercise; HIIT: high intensity interval training; M: male; F: female; BMI: body mass index.

**Table 3:** Outcome measures.

	PLA		CONT			HIIT				*P* (group × time)
	Baseline	Post	Baseline	Post	ES (95% CI)	Baseline	Post	ES (95% CI)	ES (95% CI) HIIT versus CONT	
	Body composition (DEXA)									
Trunk fat (g) Trunk fat (%)	16562.1 (1203.6) 44.6 (2.3)	17073.4 (1290.7) 45.6 (2.5)	17337.5 (742.1) 45.4 (1.7)	16627.8 (721.8) 44.2 (1.9)	−0.46 (−1.25, 0.34)	16142.5 (1153.2) 43.8 (1.5)	15840.5 (1158.5) 43.5 (1.6)	0.64 (−0.17, 1.44)	−0.79 (−1.59, 0.01)	0.70
Android fat (g) Android fat (%)	2724.0 (215.0) 48.2 (2.1)	2874.5 (259.8) 49.2 (2.2)	2884.5 (135.3) 49.8 (1.5)	2736.3 (112.3) 48.6 (1.7)	−0.32 (−1.11, 0.47)	2791.2 (184.7) 48.7 (1.3)	2736.6 (200.7) 48.7 (1.3)	0.86 (0.04, 1.68)	−0.91 (−1.72, −0.10)	0.04**
Gynoid fat (g) Gynoid fat (%)	5792.1 (306.9) 49.4 (2.1)	5776.0 (321.3) 49.3 (2.5)	6273.0 (375.3) 49.2 (2.4)	6138.1 (424.1) 48.1 (2.9)	−0.59 (−1.39, 0.21)	5540.3 (391.2) 47.6 (2.0)	5468.8 (442.9) 46.7 (2.3)	−0.34 (−1.13, 0.45)	−0.28 (−1.05, 0.58)	0.28
Total body fat (g) Total body fat (%)	31432.8 (2064.9) 42.8 (2.3)	32131.8 (2370.2) 43.4 (2.7)	33134.1 (1669.6) 43.2 (2.0)	32114.91 (184.0) 42.2 (2.3)	−0.64 (−1.45, 0.16)	30421.5 (2087.9) 41.5 (1.7)	30229.1 (2408.9) 41.0 (2.0)	0.22 (−0.57, 1.00)	−0.73 (−1.52, 0.07)	0.049**
Total lean body mass (g)	42374.8 (2892.6)	42516.5 (3531.1)	43637.8 (1873.8)	44205.2 (2364.3)	0.36 (−0.43, 1.15)	42211.6 (1360.7)	42787.9 (1608.8)	0.35 (−0.44, 1.14)	0.05 (−0.72, 0.82)	0.89

	Anthropometrics									
Weight (kg)	77.2 (3.5)	78.6 (3.8)	80.7 (1.5)	79.9 (1.8)	−0.52 (−1.32, 0.28)	76.1 (2.7)	76.3 (3.0)	0.21 (−0.58, 1.00)	−0.84 (1.64, 0.04)	0.30
Waist circumference (cm)	90.9 (3.1)	90.1 (3.2)	90.8 (2.1)	87.2 (1.7)	0.24 (−0.55, 1.03)	92.4 (2.5)	90.6 (2.5)	0.27 (−0.52, 1.06)	−0.05 (−0.82, 0.72)	0.76
Hip circumference (cm)	108.0 (1.6)	108.8 (1.7)	108.7 (1.4)	107.5 (1.5)	0.19 (−0.60, 0.97)	104.9 (2.1)	106.2 (1.9)	0.25 (−0.54, 1.04)	−0.13 (−0.9, 0.64)	0.53

	Fitness									
Work peak (W)	114.4 (16.6)	118.4 (17.5)	128.8 (10.2)	160.2 (12.3)	1.41 (0.53, 2.29)	134.7 (8.5)	164.7 (10.8)	1.17 (0.32, 2.01)	0.49 (−0.29, 1.28)	<0.001**
VO_2peak_ (mL/kg/min)*	22.3 (1.8)	22.3 (1.7)	24.0 (1.2)	28.3 (1.5)	1.63 (0.72, 2.53)	25.3 (10.4)	30.4 (1.4)	1.76 (0.83, 2.68)	0.23 (−0.54, 1.00)	<0.001**

	Biochemistry									
AST (U/L)	23.5 (2.4)	18.7 (1.0)	21.9 (2.1)	20.1 (1.2)	−0.14 (−0.92, 0.65)	27.3 (3.9)	22.5 (1.8)	−0.27 (−1.06, 0.51)	0.12 (−0.65, 0.89)	0.24
ALT (U/L)	26.7 (5.5)	17.6 (2.5)	22.0 (4.5)	17.0 (1.8)	−0.12 (−0.91, 0.66)	36.7 (8.8)	24.2 (4.8)	−0.23 (−1.02, 0.56)	0.08 (−0.69, 0.85)	0.22
Fasting glucose (mmol/L)	4.1 (0.2)	4.3 (0.1)	4.3 (0.1)	4.4 (0.1)	−0.45 (−1.24, 0.35)	4.4 (0.2)	4.3 (0.1)	−0.32 (−1.11, 0.47)	−0.19 (−0.96, 0.58)	0.41
Insulin (mU/L)	7.3 (0.7)	8.7 (1.2)	8.6 (1.4)	7.9 (1.1)	−0.39 (−1.18, 0.00)	8.0 (1.1)	7.4 (0.7)	−0.47 (−1.26, 0.33)	0.12 (−0.65, 0.89)	0.31
hs-CRP (mg/L)	1.9 (0.4)	1.7 (0.3)	4.0 (1.6)	2.9 (0.8)	−0.49 (−1.28, 0.31)	3.4 (0.8)	3.6 (1.3)	−0.71 (−1.52, 0.10)	0.03 (−0.74, 0.79)	0.45

	Lipids									
Total cholesterol (mmol/L)	6.1 (0.2)	5.4 (0.2)	5.3 (0.3)	5.5 (0.2)	−1.55 (−2.44, −0.65)	5.4 (0.3)	5.4 (0.2)	−1.46 (−2.34, −0.58)	0.25 (−1.02, 0.53)	0.02**
HDL (mmol/L)	1.6 (0.1)	1.5 (0.1)	1.4 (0.1)	1.4 (0.1)	−0.21 (−1.00, 0.57)	1.7 (0.2)	1.7 (0.2)	−0.59 (−1.39, 0.21)	0.46 (−0.32, 1.24)	0.20
LDL (mmol/L)	4.0 (0.1)	3.4 (0.1)	3.3 (0.3)	3.6 (0.2)	−1.70 (−2.61, −0.78)	3.2 (0.2)	3.2 (0.2)	−1.21 (−2.06, −0.35)	−0.38 (−1.16, 0.39)	0.02**
Triglycerides (mmol/L)	1.2 (0.1)	1.1 (0.1)	1.2 (0.2)	1.1 (0.1)	−0.30 (−1.09, 0.49)	1.3 (0.1)	1.3 (0.2)	−0.39 (−1.18, 0.40)	0.20 (−0.57, 0.97)	0.40
Free fatty acids (umol/L)	318.9 (34.5)	484.6 (106.5)	426.6 (42.0)	460.6 (54.1)	−0.89 (−1.71, −0.06)	415.6 (47.9)	493.8 (119.4)	−0.34 (−1.13, 0.45)	−0.48 (−1.26, 0.30)	0.71

	Blood pressure									
SBP (mmHg)	117.7 (5.3)	116.5 (4.7)	122.8 (4.6)	118.5 (3.2)	0.32 (−0.47, 1.11)	116.2 (3.9)	110.6 (3.2)	0.81 (0.00, 1.63)	−0.27 (−1.04, 0.50)	0.10
DBP (mmHg)	74.3 (2.5)	73.7 (2.8)	78.4 (2.8)	74.9 (2.5)	1.00 (0.17, 1.84)	76.8 (2.3)	73.9 (2.4)	0.61 (−0.19, 1.42)	0.25 (−0.52, 1.02)	0.13

Presented as Mean (SE). PLA: placebo; CONT: continuous aerobic exercise; HIIT: high intensity interval training; DEXA: dual-energy X-ray absorptiometry; VO_2peak_: peak aerobic capacity; AST: aspartate aminotransferase; ALT: alanine aminotransferase; hs-CRP: high sensitivity C-reactive protein; HDL-C: high density lipoprotein cholesterol; LDL-C: low density lipoprotein cholesterol; SBP: systolic blood pressure; DBP: diastolic blood pressure. *Estimated from *W*_peak_, *n* = 12/13/13 for PLA/CONT/HIIT, respectively, at baseline, 10/11/11 for PLA/CONT/HIIT, respectively, at postintervention testing for body composition (DEXA), and 11/11/11 for PLA/CONT/HIIT, respectively, at postintervention testing for anthropometrics, fitness, biochemistry, lipids, and blood pressure. **Significant group × time interaction (*P* < 0.05). ES: Hedge's bias corrected effect size; CI: confidence interval.

**Table 4:** Habitual energy intake and energy expenditure.

	PLA		CONT		HIIT		*P*
	Baseline	Postintervention	Baseline	Postintervention	Baseline	Postintervention	
Energy intake							
Fat (g/day)	92 (11)	78 (8)	101 (12)	87 (8)	86 (11)	79 (11)	0.80
% intake	32 (3)	30 (1)	35 (2)	34 (1)	33 (3)	33 (2)	0.98
Carbohydrate (g/day)	265 (21)	238 (14)	268 (23)	252 (22)	227 (13)	209 (20)	0.28
% intake	43 (2)	42 (2)	43 (3)	42 (2)	41 (2)	41 (2)	0.80
Protein (g/day)	116 (10)	111 (13)	108 (10)	98 (10)	103 (11)	95 (8)	0.67
% intake	20 (1)	20 (2)	17 (1)	18 (1)	19 (1)	19 (1)	0.46
Total energy intake (kJ/day)	10319 (893)	9283 (578)	10523 (917)	9527 (830)	9403 (776)	8190 (867)	0.44
Energy expenditure							
Total energy expenditure (kJ/day)	10354 (609)	9990 (557)	10527 (462)	9989 (521)	10414 (368)	10506 (450)	0.58
Steps/day	9897 (1271)	7243 (1240)	9740 (1036)	8374 (956)	9212 (820)	8989 (890)	0.42
Sedentary time, <3 METS (hours/day)	20:19 (0:58)	18:57 (1:31)	21:51 (0:11)	20:54 (1:09)	20:47 (0:41)	21:27 (0:01)	0.11
Moderate activity, 3–6 METS (hours/day)	1:29 (0:13)	1:17 (0:15)	1:25 (0:12)	1:13 (0:13)	1:31 (0:07)	1:41 (0:01)	0.57
Total self-reported energy expenditure (Bouchard score, kJ/Kg/day)	13.8 (0.6)	14.2 (0.8)	13.7 (0.4)	13.7 (0.4)	13.4 (0.3)	13.6 (0.4)	0.79

Presented as mean (SE). PLA: placebo; CONT: continuous aerobic exercise; HIIT: high intensity interval training; METS: metabolic equivalents, *n* = 12/13/13 for PLA/CONT/HIIT, respectively, at baseline, *n* = 9/10/10 for PLA/CONT/HIIT, respectively, at postintervention testing for energy intake, *n* = 10/10/11 in PLA/CONT/HIIT, respectively, at postintervention testing for energy expenditure, and *n* = 9/10/10 in PLA/CONT/HIIT, respectively, at postintervention testing for total self-reported energy expenditure.

## Abbreviations

HIIT: High intensity interval training
CONT: Continuous aerobic exercise
PLA: Placebo
*W*_peak_: Peak work capacity
DEXA: Dual-energy X-ray absorptiometry
GLUT-4: Glucose transporter type 4
BMI: Body mass index
ECG: Electrocardiogram
RPE: Rating of perceived exertion
VO_2peak_: Peak rate of oxygen consumption
ROI: Region of interest
SST: Serum separation tube
TAG: Triglyceride
TC: Total cholesterol
HDL-C: High density lipoprotein cholesterol
LDL-C: Low density lipoprotein cholesterol
ALT: Alanine aminotransferase
AST: Aspartate aminotransferase
hs-CRP: High sensitivity C-reactive protein
FFA: Free fatty acid
SBP: Systolic blood pressure
DBP: Diastolic blood pressure
HR_max⁡_: Maximal heart rate.
